# Intra-mammary lymph nodes, an overlooked breast cancer prognostic tool?

**DOI:** 10.1186/s12957-021-02219-0

**Published:** 2021-04-13

**Authors:** Tarek Hashem, Ahmed Abdelmoez, Ahmed Mohamed Rozeka, Hazem Abdelazeem

**Affiliations:** 1 Surgical Oncology Department, National Cancer Institute-Cairo University, Kasr el Aini street, Cairo, Egypt; 2 Breast Service, Shefaa Al Orman Cancer Hospital, Luxor, Egypt; 3 Radiology Department, National Cancer Institute-Cairo University, Cairo, Egypt; 4 Pathology Department, National Cancer Institute-Cairo University, Cairo, Egypt

**Keywords:** Intra-mammary lymph nodes, Breast cancer, Axillary lymph nodes

## Abstract

**Background:**

Due to the high variability of incidence and prevalence of intra-mammary lymph nodes (IMLNs), they might be overlooked during clinical and radiological examinations. Properly characterizing pathological IMLNs and detecting the factors that might influence their prevalence in different stages of breast cancer might aid in proper therapeutic decision-making and could be of possible prognostic value.

**Methods:**

Medical records were reviewed for all breast cancer patients treated at the National Cancer Institute of Cairo University between 2013 and 2019. Radiological, pathological, and surgical data were studied.

**Results:**

Intra-mammary lymph nodes were described in the final pathology reports of 100 patients. Five cases had benign breast lesion. Three cases had phyllodes tumors and two cases had ductal carcinoma in situ (DCIS). All ten cases were excluded. The remaining 90 cases all had invasive breast cancer and were divided into two groups: one group for patients with malignant IMLNs (48) and another for patients with benign IMLNs (42). Pathological features of the malignant IMLN group included larger mean tumor size in pathology (4.7 cm), larger mean size of the IMLN in pathology (1.7 cm), higher incidence of lympho-vascular invasion (65.9%), and higher rate of extracapsular extension in axillary lymph nodes (57.4%). In addition, the pathological N stage was significantly higher in the malignant IMLN group.

**Conclusion:**

Clinicians frequently overlook intra-mammary lymph nodes. More effort should be performed to detect them during preoperative imaging and during pathological processing of specimens. A suspicious IMLN should undergo a percutaneous biopsy. Malignant IMLNs are associated with advanced pathological features and should be removed during surgery.

## Background

Intra-mammary lymph nodes (IMLNs) are lymph nodes surrounded completely by breast tissue, either fatty or fibroglandular tissue, and histologically show the presence of terminal duct lobular units (TDLUs) and possibly other proliferative breast lesions as fibrocystic disease, radial scar, etc. in their vicinity. These features distinguish them from low-lying axillary lymph nodes (AxLNs) which are surrounded by axillary fat tissue [1].

IMLNs have received little attention compared to AxLNs as potential prognostic indicators in breast carcinoma. This is probably due to the relatively small number of reported cases and the rarity of studies that have focused on IMLNs.

Although IMLNs can be located in any part of the breast, they are most commonly found in the upper outer quadrant (UOQ). The prevalence of IMLNs has been reported to range between 1 and 28% [2]. Due to the high variability of the prevalence of IMLNs, they are sometimes overlooked during clinical and radiological examinations. Some authors believe that IMLNs have no clinical significance unless they are infiltrated by breast cancer. Their clinical implications in this case remain controversial [1].

Reported pathological affections of IMLNs include malignant conditions as metastatic carcinoma of a clinically evident or occult breast carcinoma and non-Hodgkin’s lymphoma.

Other inflammatory conditions such as tuberculosis have also been reported [3].

They are noted in approximately 5% of patients undergoing routine mammography.

At the National Cancer Institute of Cairo University, the Radiology Department records show a description of IMLN in 418 out of 7100 diagnostic sono-mammography examinations performed in 2019, assuming a percentage of 5.9%.

According to the current 7th edition of the American Joint Committee on Cancer AJCC staging system, there is no distinction between axillary and IMLNs, and for staging, they are considered axillary lymph nodes. Patients with IMLN metastases confirmed pathologically (excluding nodal micro-metastases) are considered to be in pathological stage II disease and are described as having positive regional metastasis even if axillary nodes are free. Therefore, the presence of IMLN metastases can upstage the disease and change therapeutic decisions [4].

On the other hand, considerable attention has been paid to the significance of extra-axillary lymph node metastases during sentinel lymph node biopsy (SLNB). Several reports describe the identification of IMLNs as the sentinel node on lymphoscintigraphy in 0.7 to 14% of patients undergoing SLNB [5].

According to MD Anderson Cancer Center experience, disease-free survival (DFS) and overall survival (OS) were significantly affected in breast cancer patients with IMLN metastases whether isolated or associated with axillary node involvement [2]. On the other hand, some reports have found that IMLN-positive/AxLN-negative patients have better prognosis than IMLN-negative/AxLN-positive patients [6].

At mammography, IMLNs can be seen as oval or round well-circumscribed homogenous densities usually smaller than 1 cm. They typically have a central lucent hilum, seen as a lower density center. On ultrasonography, they are well circumscribed, homogenously hypoechoic with mild posterior acoustic enhancement and an echogenic line representing the hilum [7]. On the other hand, suspicious IMLNs on mammography show change in normal morphology, increased size, spiculated margins, loss of the lucent center, and increased density and may have micro-calcifications within. Meanwhile, on ultrasound, they show marked hypoechogenity and thickening of the cortex, either focal or diffuse. Also, they show alterations in the central echogenic hilum with peripheral instead of hilar vascularity [8].

A current debate is ongoing, whether to perform complete axillary LN dissection (CALND) in cases that present with positive intra-mammary sentinel lymph node (IMSLN) and negative axillary SLN (AxSLN). Some studies believe that positive IMLNs do not necessarily predict AxLN metastasis and that the decision on axillary surgical management should be individualized. They suggested performing level I ALND for the management of the axilla when an IMSLN is positive, with no axillary SLN detection [9]. These studies state that management of the axilla should rely only on AxSLN status; therefore, if the IMSLN is positive and the AxSLN is detected and negative, they considered that complete ALND could be spared [9–11].

Other studies concurred that an IMSLN could act as a real SLNB. The high correlation of metastases between IMLNs and AxLNs in their results supported this opinion [12]. These studies reported that a CALND should be recommended for patients with positive IMLNs. This decision was based on findings that patients with IMLNs had more aggressive cancers as well as higher rates of lympho-vascular invasion and axillary nodal disease and higher grade and stage of disease [13, 14].

The purpose of this study is to correlate between radiological and postoperative pathological results in the assessment of intra-mammary lymph nodes. Also, to convey the importance of properly diagnosing suspicious intra-mammary lymph nodes during a radiological assessment, the sensitivity of radiological diagnosis and the effect of neoadjuvant chemotherapy on IMLN were assessed. Another aim is to highlight the effect of IMLN metastases on surgical decision and its prognostic value in breast cancer patients, in order to improve diagnosis and management of malignant IMLN in breast cancer.

## Patients and methods

This is a retrospective study focusing on the pathological, radiological, and clinical features of IMLNs. The records were reviewed for all breast cancer patients treated at the National Cancer Institute of Cairo University between 2013 and 2019.

Inclusion criteria were patients with full records, who have shown the presence of IMLNs on their pathology report. The clinical, radiological, and surgical data have been studied. A comparison was done between patients with pathologically positive IMLNs for breast cancer metastases and those with negative IMLNs to determine possible effects of patient and tumor factors on the probability of IMLN metastases. A correlation between the status of IMLNs and axillary lymph nodes was also sought.

IMLN radiological diagnosis was the ability of ultrasonography to diagnose the IMLN as either a mass, likely IMLN, definitive diagnosis of IMLN, or not identified, whereas IMLN radiological criteria were the diagnosis of the IMLN/mass as either benign, suspicious, or not identified.

If the IMLN has completely lost its normal morphology in a way to appear as an irregular hypoechoic mass, then in this case it will be difficult to name it an IMLN. In this instance, it will be described in the radiological report as a mass and can only be diagnosed as an infiltrated intra-mammary lymph node by the pathologists after a biopsy has been obtained.

In the diagnosis of IMLN metastasis in a known case of breast cancer, we differentiate between a pathological lymph node and an indeterminate lymph node. An indeterminate lymph node is a lymph node with focal cortical thickening exceeding 3 mm and an eccentric hilum or showing diffusely thickened cortex. In our retrospective study, we did not find any cases who underwent tissue biopsy of IMLN in the 90 patients included (was performed in excluded patients) and FNAC was performed only in cases of indeterminate IMLNs.

In cases with bilateral lymph nodes, we described the diseased one, and in cases of bilateral breast cancer, we described the breast that has IMLN.

Patients who received neoadjuvant chemotherapy were not excluded from our study, and the decision of neoadjuvant chemotherapy was a multidisciplinary team (MDT) decision. The decision in these cases was based upon tumor biology, tumor to breast ratio in patients who desired breast conservation, and the clinical stage.

At the National Cancer Institute in Cairo University, frozen section examination is performed in cases with radiologically negative axillary nodes, who undergo SLN. According to the SLN result, complete axillary clearance is performed or spared. An IMLN is considered as an axillary lymph node, and when excised as IMSLN, it is counted to the rest of the SLN harvest.

Data were statistically described in terms of mean ± standard deviation (±SD), and/or frequencies (number of cases) and percentages when appropriate. Comparison of numerical variables between the study groups was done using the Student *t* test for independent samples in normally distributed data and the Mann–Whitney *U* test for independent samples in not normal data. For comparing categorical data, the chi-square (*χ*^2^) test was performed. An exact test was used instead when the expected frequency is less than five. *P* values less than 0.05 were considered statistically significant. All statistical calculations were performed using computer program SPSS (Statistical Package for the Social Science; SPSS Inc., Chicago, IL, USA) release 15 for Microsoft Windows (2006).

## Results

IMLNs were described in the postoperative pathological specimens of 100 patients. Five cases did not suffer from breast cancer; they underwent wide excision of suspicious masses, which revealed to be benign. Three other cases had malignant phyllodes for which they underwent mastectomy. Another two cases had ductal carcinoma in situ (DCIS). All ten cases were excluded. The remaining 90 patients all had a final pathological diagnosis of invasive breast carcinoma and included IMLNs in their specimens. The mean age of the study group was 48.2 ± 10.2 years.

Overall, pathology reports showed that 42 (46.7%) cases had benign IMLNs, while 48 cases (53.3%) had malignant deposits in IMLNs. Accordingly, patients were categorized into two groups: one group for patients with malignant IMLNs and another for patients with benign IMLNs.

All cases underwent preoperative conventional sono-mammography evaluation of both breasts and axilla. The preoperative imaging was able to detect IMLN definitely in 47 cases (52.2%) and commented on them as likely IMLN in 17 cases (18.8%). In 12 (13.3%) cases, the IMLN was seen as a mass of ill-defined nature. Conventional sono-mammography failed to identify IMLN in 14 cases (15.5%). The age and radiological features of the study group are illustrated in Table 1.

**Table 1:** Age and radiological features of the study group

Variables	Benign IMLNs (***N*** = 42)	Malignant IMLNs (***N*** = 48)	***P***-value
**Age in years,** mean ± SD	46.7 ± 10.2	49.54 ± 10.1	0.19^A^
**Laterality, no. (%)**			
Left	24 (57.1%)	21 (43.8%)	
Right	17 (40.5%)	26 (54.2%)	0.42^C^
Bilateral	1 (2.4%)	1 (2.1%)	
**Tumor site, no. (%)**			
Central	6 (14.3%)	7 (14.6%)	
LIQ	7 (16.7%)	2 (4.2%)	0.32^C^
LOQ	3 (7.1%)	1 (2.1%)	
Multicentric	13 (31%)	23 (8.9%)	
UIQ	4 (9.5%)	4 (8.3%)	
UOQ	9 (21.4%)	11 (22.9%)	
**No. of IMLNs in radiology, no. (%)**			
0	9 (21.4%)	17 (35.4%)	
1	29 (69%)	24 (50%)	
2	1 (2.4%)	5 (10.4%)	0.14^C^
3	3 (7.1%)	2 (4.2%)	
**IMLN radiological diagnosis**			
**Definitive**	24 (57.1%)	23 (47.9%)	
**Likely**	9 (21.4%)	8 (16.7%)	0.46^C^
**Not identified as IMLN**	5 (11.9%)	7 (14.6%)	
**Not identified at all**	4 (9.5%)	10 (20.9%)	
**IMLN radiological criteria**			
Benign	7 (16.7%)	3 (6.3%)	
Not identified	4 (9.5%)	10 (20.8%)	0.13^C^
Suspicious	31 (73.8%)	35 (72.9%)	
**IMLN site radiologically, no. (%)**			
LIQ	1 (2.4%)	1 (2.1%)	
LOQ	1 (2.4%)	5 (10.4%)	0.319^C^
UIQ	6 (14.3%)	4 (8.3%)	
UIQ LOQ	1 (2.4%)	0	
UOQ	27 (64.3%)	27 (56.3%)	
UOQ UIQ	0	1 (2.1%)	
UOQ LOQ	1 (2.4%)	0	
UOQ UIQ	1 (2.4%)	0	
**IMLN site radiologically, no.**			
**UOQ**	29	28	0.93^C^
**Non-UOQ**	9	10	
**IMLN in other breast**	4 (9.5%)	2 (4.2%)	0.277^C^
**Preoperative FNAB of IMLN**	9 (20.4%)	10 (20.8%)	0.51^C^
**FNAB of IMLN findings**			
Benign	2 (4.8%)	0	
Inconclusive	2 (4.8%)	1 (2.1%)	0.066^C^
Suspicious	5 (11.9%)	6 (12.5%)	
Metastatic	0	3 (6.3%)	
**Axillary LN by ultrasound**			
Non-specific	6 (14.3%)	2 (4.2%)	0.095^C^
Pathological	36 (85.7%)	46 (95.8%)	

^A^Unpaired *t* test, ^B^Mann–Whitney, ^C^chi-square

The sonographic features of detected IMLNs were seen as benign in 10 (11.1%) cases and as suspicious IMLN or mass in 66 cases (73.3%). Pathology reports revealed that 31 of the suspicious IMLNs were benign, while 35 proved to be malignant.

Thirteen cases in each group received neoadjuvant chemotherapy. The response to chemotherapy in axillary lymph nodes (AxLNs) and IMLNs was described using Sataloff’s classification. Miller–Payne grading system determined the response of the primary breast tumor to neoadjuvant chemotherapy with grade 1 indicating no change in tumor up to grade 5 showing pathological complete response (no malignant cells at the site of tumor) (Tables 2 and 3).

**Table 2:** Patients who received neoadjuvant chemotherapy in each group

Variables	Benign IMLNs Received Neoadj CTH (***N*** = 13)	Malignant IMLNs Received Neoadj CTH (***N*** = 13)	***P***-value
**Neoadjuvant chemotherapy**			
**Yes**	13 (31%)	13 (27.1%)	0.686^C^
**No**	29 (69%)	35 (72.9%)	
**AxLN response after neoadjuvant chemotherapy**			
**N-A**	6 (46.2%)	4 (30.8%)	0.199^C^
**N-B**	1 (7.7%)	0 (0.0%)	
**N-C**	3 (23.1%)	1 (7.7%)	
**N-D**	3 (23.1%)	8 (61.5%)	
**IMLN response after neoadjuvant chemotherapy**			
**N-A**	12 (92.3%)	0 (0%)	< 0.001^C^
**N-B**	1 (7.7%)	0 (0%)	
**N-D**	0.(0%)	13 (100%)	
**Miller grade tumor**			
**1**	0 (0%)	3 (23.1%)	
**2**	3 (23.1%)	7 (53.8%)	
**3**	1 (7.7%)	0 (0%)	
**4**	1 (7.7%)	1 (7.7%)	0.020^C^
**5**	8 (61.5%)	2 (15.4%)	

^A^Unpaired *t* test, ^B^Mann–Whitney, ^C^chi-square

**Table 3:** Diagnostic accuracy of ultrasound in the diagnosis of IMLN metastases

Variables	US findings			
	Benign		Suspicious	
	No.	%	No.	%
Pathological findings				
Benign	6	24	1	4
Malignant	19	76	24	96
Total	25		25	
Sensitivity of US	96% CI (79.65 to 99.90%)			
Specificity	24% CI (9.36 to 45.13%)			
PPV	55.8% CI (49.98 to 61.49%)			
NPV	85.7% CI (43.75 to 97.89%)			
Accuracy	60% CI (45.18 to 73.59%)			

Here, we tried to assess the sensitivity and specificity of ultrasound in the diagnosis of malignant IMLNs. In this table only, we excluded patients who received neoadjuvant chemotherapy to exclude the liability of the therapy effect of chemotherapy on the final pathological status of IMLNs.

Pathological features of the malignant IMLN group included a larger mean tumor size (4.7 cm), a larger mean size of the IMLN (1.4 cm), a higher incidence of lympho-vascular invasion (65.9%), and a higher rate of extracapsular extension in axillary lymph nodes (57.4%). All these criteria were statistically significant. In addition, the pathological (N) stage was significantly higher in the malignant IMLN group.

In thirteen cases out of 48 (27.6%), positive IMLNs were associated with negative axillary lymph nodes with 4 out of the 13 patients received neoadjuvant chemotherapy. On the other hand, 20 cases out of 42 (47.6%) had a benign IMLN with positive axillary lymph node metastasis. The pathological features of both study groups are highlighted in Table 4.

**Table 4:** Pathological features of both groups

Variables	Benign IMLNs (***N*** = 42)	Malignant IMLNs (***N*** = 48)	***P***-value
**Type of breast surgery**			
Breast conservative surgery	6 (14.3%)	6 (12.5%)	1^C^
Mastectomy	36 (85.7%)	42 (87.5%)	
**Type of axillary surgery**			
**SLNB**	6 (14.2%)	3 (6.25%)	
**Axillary clearance**	36 (85.7%)	44 (91.6%)	0.29^C^
**No surgery**	0	1 (2%)	
**Tumor pathology, no. (%)**			
Invasive duct carcinoma	37 (88.1%)	45 (93.7%)	
Invasive lobular carcinoma	4 (9.5%)	3 (6.2%)	0.56^C^
Mucinous carcinoma	1 (2.3%)	0 (0%)	
**Hormone receptor status**			
Luminal A	18 (42.8%)	24 (50%)	
Luminal B	5 (11.9%)	7 (14.5%)	0.45^C^
Luminal Her2	5 (11.9%)	9 (18.7%)	
Her 2 enriched	8 (19%)	5 (10.4%)	
Triple-negative breast cancer	6 (14.2%)	3 (6.2%)	
**Associated DCIS, no. (%)**	14 (33.3%)	20 (41.7%)	0.43^C^
**Pathological tumor size (cm)**			
Mean ± SD	3.3 ± 1.2	4.7 ± 2.6	0.001^B^
**No. of IMLNs**			
Mean ± SD	1.2 ± 0.61	1.4 ± 0.84	0.377^B^
**Pathological IMLN size**			
Mean ± SD	1.1 ± 0.2	1.7 ± 0.6	0.001^B^
**Lympho-vascular invasion, no. (%)**			
Yes	6 (14.3%)	31 (65.9%)	0.001^C^
No	36(85.7%)	16 (34%)	
**IMLN site pathologically, no. (%) (specified)**			
Central	0	2 (4.2%)	
LIQ	1 (2.4%)	1 (2.1%)	
LOQ	1 (2.4%)	4 (8.3%)	0.48^C^
UIQ	6 (14.3%)	7 (14.6%)	
UOQ	32 (76.2%)	33 (68.8%)	
UOQ UIQ LOQ	1 (2.4%)	0	
UOQ LOQ	1 (2.4%)	0	
UOQ UIQ	0	1 (2.1%)	
**IMLN site pathologically, no. (%) (collected)**			
**UOQ**	34	34	
**Non-UOQ**	8	14	0.265^C^
**Extracapsular extension AxLNs, no. (%)**			
Yes	7 (16.7%)	27 (57.4%)	0.001^C^
No	35 (83.3%)	21 (44.6%)	
**Pathological T stage**			
T1	2 (4.8%)	3 (6.3%)	
T2	34 (81%)	22 (45.8%)	0.006^C^
T3	4 (9.5%)	13 (27.1%)	
T4	2 (4.8%)	10 (20.8%)	
**Pathological N stage for axilla only**			
Nx	0	1	
N0	22 (52.4%)	13 (27.6%)	0.002^C^
N1 1–3	13 (31%)	9 (19.1%)	
N2 4–9	7 (16.7%)	14 (29.7%)	
N3 ≥ 10	0	11 (23.4%)	
**Pathological N stage including IMLN**			
Nx	0	1	
N0	22 (52.4%)	0	0.002^C^
N1 1–3	13 (31%)	22 (46.8%)	
N2 4–9	7 (16.7%)	14 (29.7%)	
N3 ≥ 10	0	11 (23.4%)	

^A^Unpaired *t* test, ^B^Mann–Whitney, ^C^chi-square

## Discussion

IMLNs may be overlooked during breast imaging and during specimen processing in cases of breast cancer. They might not be excised in cases of breast-conserving surgery. Thus, it is important to define the significance and the incidence of metastasis in these nodes.

In cases of early-stage breast cancer, the identification of IMLN metastases in a patient with otherwise negative AxLNs not only will result in upstaging but also may alter adjuvant therapy planning. This study indicates that malignant IMLN seems to be associated with aggressive and advanced disease.

In our practice, if the IMLN is highly suspicious in sono-mammography, we do not do percutaneous biopsy of the node and manage it as a malignant node that should be excised. We spare a biopsy for indeterminate nodes either axillary or IMLN. Even if the biopsy for highly suspicious IMLN is performed and the result is negative, we localize the IMLN by wire or radioactive material and remove it during surgery.

The IMLNs are not accurate indicators of axillary lymph node status. In this study, almost 30% (27.6%) of patients with malignant IMLN had negative axillary lymph nodes. This comes in concordance with previous reports observing that one third of patients with positive IMLN have free axillary nodes [15]. On the other hand, this study shows a considerable percentage of patients with axillary lymph node metastasis to have benign IMLN (47.6%). This is higher than previous reports of only 15% [15].

At our center, during management of patients with only IMLN metastases and no suspicious AxLNs, we excise the IMLN and SLN and send them for frozen section. If the result of the frozen section shows only one positive node out of ≥ 3 negative nodes, even if the positive node is the IMLN, and also in case or the IMLN and SLNs are negative, we do not perform axillary clearance. Therefore, we consider the IMLN as an AxLN and excise it only when it is suspicious radiologically, malignant by preoperative biopsy, or if it is the SLN.

IMLNs have proved to be a separate possible site of metastases in breast cancer. Preoperative lymphoscintigraphy may help identify these extra-axillary metastases and thus control them during management.

IMLNs sometimes are suspicious radiologically, but still have a negative result on biopsy, and in these cases, our practice is to localize and excise the suspicious lesion during surgery. This study showed that in some cases a change of the surgical decision from breast conservation to mastectomy took place, based on the suspicion of multicentricity. However, the lesion turned out to be an IMLN rather than a multicentric disease.

Thus, meticulous radiological, pathological, and clinical attention should be given to IMLN detection for its possible direct effect on therapeutic planning.

Increased care should be taken, and the scope of suspicion should be widened during preoperative radiological evaluation.

If a suspicious IMLN is found, percutaneous biopsy should be performed in order to verify its status. Data suggest that it should not be regarded as an indicator for axillary lymph node status. Moreover, a thorough clinical evaluation of the axilla is warranted in every patient.

Incidence of IMLN detection is much higher in mastectomy specimens rather than BCS. This is explained by the fact that BCS is a tumor-focused procedure, while mastectomy specimens include neoplastic and non-neoplastic findings as benign and other breast lesions. Another limitation in BCS is when the tumor is located at UOQ or axillary tail and excised en bloc with the axilla. In such situation, IMLN may be interpreted as one of the axillary LNs.

IMLNs may be mistaken for malignant masses radiologically and commented upon as a tumor satellite or multicentric breast cancer. For a definitive differentiation between malignant IMLNs and malignant masses in the pathological specimen, the IMLNs are surrounded by breast tissue (TDLUs) and show lymphoid tissue surrounded by capsule with macro-metastases. This may involve part or most of the IMLN with a residual rim of nodal lymphoid tissue, or completely replace the lymphoid tissue with only preserved IMLN capsule and occasional lymphoid foci if heavily infiltrated.

This study has several limitations. First, the retrospective study design mandates careful evaluation of the results. In addition, the small study population is also a weak point. There is a degree of bias regarding the true incidence of IMLNs due to two factors. First, the IMLN may not be excised during breast-conserving surgery. Second, the routine pathological processing of specimens might overlook an excised IMLN.

Despite these limitations, this study is one of the largest to evaluate IMLNs and their relation to patient and tumor factors. To overcome the study’s shortcomings, a multicenter prospective study involving a larger cohort with long-term follow-up is necessary to define the true significance and prognosis of IMLN.

## Conclusion

Clinicians frequently overlook intra-mammary lymph nodes. More effort should be performed to detect them during preoperative imaging and during pathological processing of specimens. A suspicious IMLN should be biopsied. Malignant IMLNs are associated with advanced pathological features and should be removed during surgery, which may change the surgical decision.

## Data Availability

Please contact the author for data request.
